# EVALUATING THE EFFECTIVENESS OF AN ONLINE TRAINING COURSE FOR MEETING NEEDS ASSOCIATED WITH CAREGIVING BURDEN

**DOI:** 10.1093/geroni/igac059.1720

**Published:** 2022-12-20

**Authors:** Shera Hosseini, Lorraine Carter, Donna Thomson, Michelle Howard

**Affiliations:** McMaster University, Hamilton, Ontario, Canada; McMaster University, Hamilton, Ontario, Canada; McMaster University, Hamilton, Ontario, Canada; McMaster University, Hamilton, Ontario, Canada

## Abstract

Informal caregivers make up a critical part of long-term support in the communities due to their important role in caring for older adults living at home. Modern-day caregivers are facing greater responsibility and burden for managing their care recipients. The present study aimed to explore the effect of a caregiving training program in alleviating caregiver burden. This program included four standalone online modules each with specific foci. Completion of activities across the modules allowed for the creation of a Caregiver Action Plan which offered a personal and practical resource to the informal caregivers. This evaluation study was qualitative and a used thematic analysis method of data analysis. Data stemmed from semi-structured interviews with the caregivers and their reflections on the program’s discussion board. Most caregivers provided care for persons with dementia. Interviews with the family caregivers were conducted, transcribed, and thematically analyzed. Themes were identified through constant comparison and in an iterative process. The family caregivers demonstrated consensus on the efficacy of the program in raising competence and confidence and contributing to ameliorating burden levels. Important themes were identified in association with areas for which the caregivers needed support: Early dementia education, planning for future care, learning about navigating healthcare systems, peer support, enhancing self-care, and coping with emotional burden and self-blame. The findings will be informative in shaping the program based on the caregiver’s identified needs through addressing those areas that they would need support. These findings may offer recommendations to other programs designed to support the family caregivers.

